# Imaging in Infective Endocarditis—Current Opinions and Trends in Cardiac Computed Tomography

**DOI:** 10.3390/diagnostics14131355

**Published:** 2024-06-26

**Authors:** Ana Petkovic, Nemanja Menkovic, Olga Petrovic, Ilija Bilbija, Miodrag Nisevic, Nikola N. Radovanovic, Dejana Stanisavljevic, Svetozar Putnik, Ruzica Maksimovic, Branislava Ivanovic

**Affiliations:** 1 Diagnostic Department of Center of Stereotaxic Radiosurgery, Clinic of Neurosurgery, University Clinical Center of Serbia, 11000 Belgrade, Serbia; nemanjam59@hotmail.com; 2 Cardiology Clinic, University Clinical Center of Serbia, 11000 Belgrade, Serbia; opetrovic1976@gmail.com; 3 School of Medicine, University of Belgrade, 11000 Belgrade, Serbia; i.bilbija@yahoo.com (I.B.); nikolar86@gmail.com (N.N.R.); svetozar073@yahoo.com (S.P.); dr.ruzica.maksimovic@gmail.com (R.M.); 4 Department for Cardiac Surgery, University Clinical Center of Serbia, 11000 Belgrade, Serbia; 5 Center of Radiology, University Clinical Center of Serbia, 11000 Belgrade, Serbia; miodrag.nisevic@gmail.com; 6 Pacemaker Center, University Clinical Center of Serbia, School of Medicine, University of Belgrade, 11000 Belgrade, Serbia; 7 Institute for Medical Statistics and Informatics, Faculty of Medicine, University of Belgrade, 11000 Belgrade, Serbia; sdejana8@yahoo.com

**Keywords:** infective endocarditis, cardiac computed tomography, transthoracic echocardiography, transesophageal echocardiography

## Abstract

Infective endocarditis is a rare disease with an increasing incidence and an unaltered high mortality rate, despite medical development. Imaging plays an integrative part in the diagnosis of infective endocarditis, with echocardiography as the initial diagnostic test. Research data in the utility of cardiac computed tomography (CCT) in the diagnostic algorithm of IE are rising, which indicates its importance in detection of IE-related lesion along with the exclusion of coronary artery disease. The latest 2023 European Society of Cardiology Guidelines in the management of IE classified CCT as class of recommendation I and level of evidence B in detection of both valvular and paravalvular lesions in native and prosthetic valve endocarditis. This review article provides a comprehensive and contemporary review of the role of CCT in the diagnosis of IE, the optimization of acquisition protocols, the morphology characteristics of IE-related lesions, the published data of the diagnostic performance of CCT in comparison to echocardiography as the state-of-art method, as well as the limitations and future possibilities.

## 1. Introduction

Infective endocarditis (IE) is the infection of cardiac native valves (NVE), prosthetic valves (PVE), and implantable electronic devices (CIED) [1,2,3].

IE is a rare disease, which remains impermanently at the center of attention [4,5,6,7]. Its incidence is rising and the mortality rate remains unaltered despite advances in the medical field [8,9]. The latest epidemiological studies indicate that age-specific death rates decreased in low-developed countries by 13.3% from 1990 to 2012, due to greater availability of antibiotics, in contrast to highly developed countries, where they increased by 71.4% between 1990 and 2007, due to changes in risk factors. Globally, the number of deaths associated with infective endocarditis was 20,754 in 1990, rising to 66,322 in 2019 [10]. The incidence of infective endocarditis has been increasing continuously and is estimated between 3 and 10 cases per 100,000 population [11]. The recent meta-analyses of population-based studies in Europe highlighted a 4.1 ± 1.2% annual increase in incidence of IE, which has doubled between 2000 and 2018 [8]. The latest research found that worldwide annual incidence in 2019 increased to 13.8 cases per 100,000 population compared to 9.91 cases per 100,000 population in 1990 [10,12,13].

Demographic changes have made a shift towards the older population with numerous comorbidities [13]. The average age of patients is between 58 and 80 years, with more frequent male population between 42% and 72% [14]. Probably, females are under the protective role of estrogen to endothelium, and use health services frequently. On the other hand, degenerative aortic and mitral valve diseases are more common in men. Mortality is higher in the male population and in the elderly due to weakened immune response, lack of extensive expertise in treatment, and weaker therapeutic response [13]. In the pediatric population, infective endocarditis is rare, but is accompanied by a high mortality rate of about 25%. There is an increase in the incidence in this age group, with 0.34 to 0.64 cases per 100,000 population, mostly as a result of the improvement in the survival rate in children with congenital heart defects (CHD) [15,16].

Risk factors have remained unchanged in countries with a lower standard, with rheumatic heart fever as the dominant risk factor [1,17]. In countries with a higher standard, the risk factors have changed towards artificial valves and endovascular implanted materials, congenital heart defects, invasive diagnostic and therapeutic procedures, degenerative valves, diabetes mellitus, oncology, hemodialysis, immunosuppressive therapy, and intravenous drug users [1,18,19,20,21,22].

Changes in microbiological agents have placed staphylococcus as the leading cause of IE in 44.1% of cases, followed by enterococcus in 15.8% of cases, especially in the elderly population [23]. Streptococcus remains the dominant cause in low-developed countries, but in highly-developed countries it is the third leading cause in 12.4% of cases [1,23]. The remaining microbes that can cause infective endocarditis are rare bacteria (HACEK group—*Haemophilus* species, *Aggregatibacter actinomycetemcomitans*, *Cardiobacterium hominis*, *Eikenella corrodens*, and *Kingella kingae*), zoonoses, or fungi [1,23,24]. Infective endocarditis with negative blood cultures is highly variable and represents a diagnostic and therapeutic problem. It is most often a consequence of previously applied antibiotic therapy or rare pathogens, such as fungi or rare bacteria, which grow relatively slowly [19].

Diagnosis of IE is based on clinical suspicion sustained by microbiological confirmation and detection of IE-related lesion by imaging modalities [1,19]. Echocardiography is the first-line and key imaging method in the diagnosis and decision-making process, in monitoring patients during medical therapy, in the peri- and postoperative period [19,25,26]. Cardiac computed tomography (CCT) has emerged having additional imaging modalities for confirmation of diagnosis in possible IE, in the detection of the extent of invasive disease, or in extracardiac dissemination [27]. Positron emission computed tomography/computed tomography (PET/CT) is recommended in possible PVE to confirm diagnosis. White blood cell single photon emission tomography/computed tomography (WBC SPECT/CT) is an alternative nuclear imaging technique, when PET/CT is unavailable and in inexperienced centers. Advantages and limitations of the single cardiovascular imaging techniques in the diagnosis of IE are summarized in Table 1.

The aim of this article is to review the role of CCT in detection of IE-related lesion. 

## 2. Role of Cardiac CT in Imaging Algorithm of Infective Endocarditis

In 2023, Duke’s International Society for Cardiovascular Infectious Diseases and the European Society of Cardiology (ESC) revised the criteria in the diagnosis and treatment of infective endocarditis, with a significant change in the role of CCT as a major criterion for detection of valvular and paravalvular IE lesions [19,28]. The 2023 ESC Guidelines classify CCT as class of recommendation I and level of evidence B in detection of both valvular and paravalvular lesions in native valve endocarditis (NVE) and prosthetic valve endocarditis (PVE) [19]. Cardiac CT is proposed as a complementary imaging method to echocardiography due to better spatial resolution. In the diagnostic algorithm, both transthoracic echocardiography (TTE) and transesophageal echocardiography (TOE) are recommended as the first-line imaging modality in suspected IE, except in right-sided NVE where quality and conclusive TTE is sufficient. In cases of possible NVE and PVE, CCT is recommended to detect valvular lesions and confirm diagnosis. In cases of definite IE with suspected paravalvular complication or inconclusive TOE, CCT is recommended in NVE and PVE [19].

**Figure 1:**
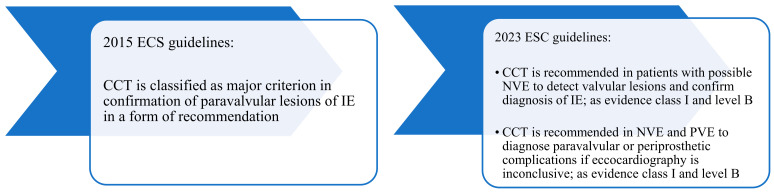
Differences in the role of cardiac CT from previous to the current ESC Guidelines.

The previous and the current role of CCT in the diagnosis of IE according to ESC Guidelines (Figure 1) [19,27]:

## 3. Technical Improvement of Cardiac Computed Tomography

The quality of the CT images depends on the spatial and temporal resolution, which is improving continuously along with technological development. Temporal resolution has improved up to 24 ms, due to acceleration of the rotation time and the appearance of dual-source X-ray tubes. Spatial resolution has improved by multiplying the rows of scintillator detectors up to 256 rows and the appearance of photon-counting detectors, enabling the direct conversion of X-rays into an electrical signal. Direct conversion increases utilization of X-rays, as opposed to scintillator detectors, which are characterized by a loss of 30% of their value during the light conversion. The spatial resolution of scintillator detectors is up to 0.23 mm, while that of photon-counting detectors is up to 0.11 mm. Considering the patient’s exposure to ionizing radiation, dose modulation protocols are used to lower the voltage and current of the X-ray tube according to the patient’s habitus. However, the reduction of ionizing radiation reduces the contrast of the image and consequently the resolution, which is improved by the application of new software tools in data processing, such as iterative reconstructions, when compared to the existing filtered back projection [29,30,31].

## 4. Cardiac CT Protocol

### 4.1. Acquisition

Electrocardiographic (ECG)-gated CCT is needed for the analysis of cardiac structure. It is advisable not to perform dose modulation protocols, as low voltage image decreases spatial resolution in the detection of IE-related lesions, regardless of image reconstruction protocols, filtered back projection (FBP) or iterative. All CT scans should be performed with three-phase biventricular contrast injection protocols. The amount of the contrast medium is determined by patient weight, being 1.5–2 mL/kg approximately. In the first phase, patients should have been administered two thirds of the entire amount of pure contrast medium. In the second phase, patients should have been administered a mixture of saline and contrast medium, in two thirds the volume of the previous phase. The proportion of contrast medium and saline, in left-sided IE should be 30% to 70% in favor of saline, whereas in right-sided IE it should be equal to 50%, at a slower rate of 3 mL/s, in order to avoid turbulent flow in the right atrium and ventricle as much as possible. The third phase consists of pure saline. The time to exposition, from intravenous administration of contrast medium, should be calculated by the time-bolus technique, with the region-of interest (ROI) positioned at the aortic root at the level of origin of the left main artery. Retrospective image data should be reconstructed at 10–90% of the R-R interval of the cardiac cycle. In a case of irregular heart rhythm additional reconstruction around 300 ms should be performed [31,32]. ECG CCT examination should be followed by non-ECG-gated examination of thorax, abdomen, and pelvis in two acquisitions. The first acquisition should start as soon as possible, and it will correspond to the late arterial phase at an approximate acquisition time around 35 s. The second acquisition should start with 30 s delay time in order to acquire the venous phase. 

### 4.2. Image Analysis

From multiplanar reformations, double-oblique reformation should be used in order to create cardiac valves planes in their longitudinal and transverse sections. Aortic and mitral valves are analyzed in the diastolic phases, but if the patient’s heart rate is above 70 beats/min, systolic phase is advisable [31,32].

## 5. Infective Endocarditis Related Lesions

Causative microorganisms adhere to the damaged areas of the endocardium or foreign material. Its growth leads to thickening of valve cusps, with consecutive development of valvular lesions: vegetations, perforations, and aneurysms. With the development of an invasive form, the disease spreads along the annulus into the extravascular space and forms paravalvular lesions: abscesses, pseudoaneurysms, fistulas, and leaks [33,34,35].

The correlation between the morphological features of IE at surgery/autopsy, echocardiography, and CCT are described in Table 2 [27,36,37,38,39,40,41].

## 6. Cardiac CT Imaging of Valvular Lesions

### 6.1. Vegetations

**Figure 2:**
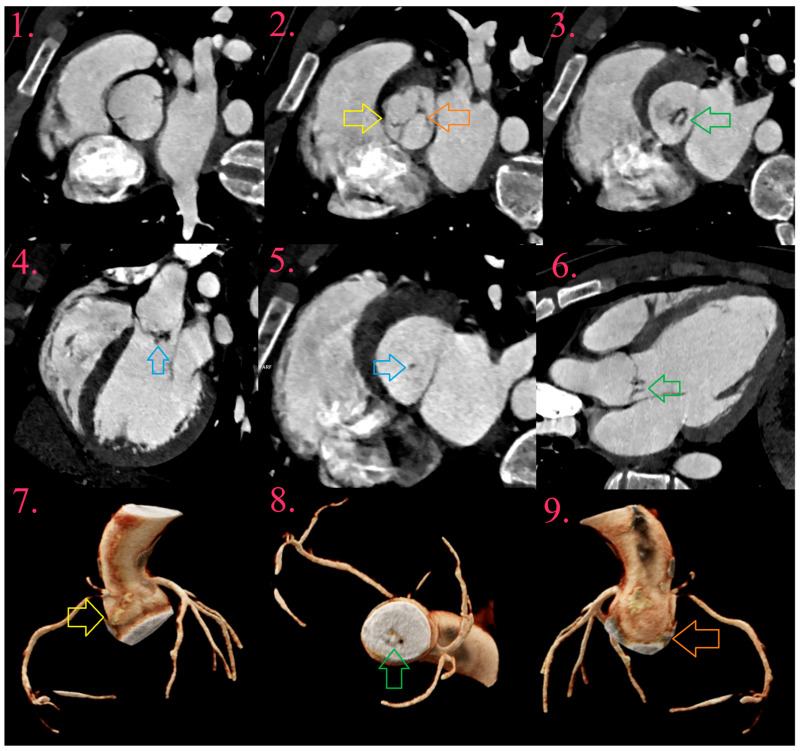
IE of fused bicuspid aortic valve (BAV) in young male patient (Image 1), with fused right and left coronary cusps (Image 2 and Image 7, yellow arrow) and non-coronary cusp (Image 2 and Image 9, orange arrow), with aneurysm development at its bottom (Image 3, Image 6, and Image 8, green arrow). There is uniform thickening of aneurysm, with small vegetation (Image 4 and Image 5, blue arrow).

**Figure 3:**
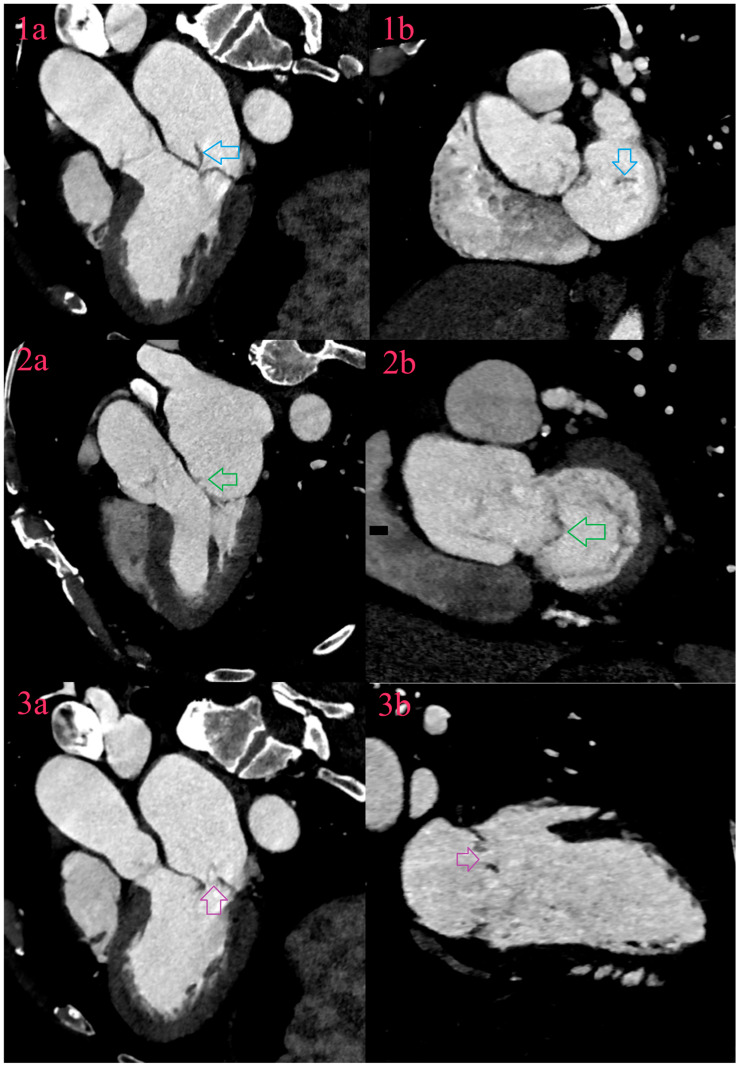
Valvular lesions of IE of mitral valve. There is vegetation arising from free margin of posterior leaflet (Image 1a and Image 1b, blue arrow), aneurysm of anterior leaflet in A2 segment (Image 2a and Image 2b, green arrow) and perforation of aneurysm on the field of prolaps of P2 segment of posterior leaflet (Image 3a and Image 3b, purple arrow).

A vegetation appears as a hypodense soft tissue lesion of variable size or focal thickening along the valve cusps or foreign material [32]. They are a basic pathoanatomic entity, which becomes visible at least two weeks after the onset of the disease. Their appearance may be in the form of a verruca or a plate. In NVE, they are localized along the blood flow surface, around the free edge of the cusps or leaflets, or in the attachment zone. Also, they are localized to the low-pressure side—to the atrial side of the mitral and tricuspid valves and to the ventricular side of the aortic and pulmonary valves. The most affected valves are the mitral, followed by the aortic, tricuspid and, least often, the pulmonary valve (Figure 2 and Figure 3) [35]. In PVE, they are usually localized in the suture ring, or on smaller thrombi in the low flow zones of the prosthesis. In biological valves, they are localized along the leaflets [42].

The key to vegetation analysis is the assessment of the embolic potential [10,11]. Size and mobility are the most important independent risk factor, along with location on the mitral valve, size change during antibiotic therapy, particular microorganisms (Staphylococcus species), previous embolism, multivalvular involvement, and biological markers. CCT may depict oscillating movement during the cardiac cycle at the cine images and if the length ≥10 mm, surgical treatment is required [19].

Sensitivity and specificity for detection of vegetations range for TTE 41–90% and 58–96%, whereas for TEE they are higher 89.3–100% and 83.3–100% [43,44,45,46,47,48,49,50,51,52,53,54,55,56,57]. Due to better temporal resolution, echocardiography can easily detect vegetation mobility, but unfavorable patient’s habitus, heavy valve calcification, and prosthetic valves may impair its visibility [44]. Adding CCT to the diagnostic algorithm improves vegetation detection. According to the heretofore available studies, CCT has sensitivity 70–96% and specificity 20–100%, as a single used test [45,46,47,48,49,50,51,52,53,54,55,56,57].

In 2009, Feuchtner et al. reported in 29 patients with 57 left-sided NVE and PVE, similar sensitivity and specificity for TEE and CCT (97% and 95% vs. 96% and 97%) [45]. In 2010, Gahide et al. published the research results of 19 patients with infective endocarditis of the aortic valve, 2 NVE and 17 PVE. The sensitivity and specificity of CCT in vegetation detection was 71.4% and 100% [46]. In 2013, Habets et al. reported in 28 patients with PVE, that adding CCT to the standard imaging protocol (TTE + TEE), raised sensitivity to 100% vs. 63% [47]. In 2017, Koo et al. conducted a study in 49 patients with infective endocarditis of 23 aortic, 13 mitral, and 2 tricuspid valves, in a ventricular septal defect and at the orifice of the inferior vena cava. Two patients had multivalvular left-sided disease and 12 NVE. In the detection of vegetation, the sensitivity of TEE was higher at 100%, while for CCT it was slightly lower at 90.9% [48]. In 2018, Ouichi et al. reported in 14 patients with left-sided infective endocarditis, sensitivity of TEE 100% and CCT 92.3% [49]. The same year, Sims et al. conducted a study in 255 patients with surgically confirmed infective endocarditis, but only 34 had CCT, while all patients underwent TEE. The sensitivity and specificity of TEE in detection of 180 vegetations was 95.6% and 93%, while for CCT in detection of 20 vegetation was 70% and 92.9% [50]. Also in 2018, Koneru et al. published the results of a study in 122 operated patients with 141 valves, both NVE and PVE. The sensitivity and specificity of TEE were for both valve types 85% and 69%, for NVE 96% and 67%, and PVE 78% and 70%. The sensitivity and specificity of CCT were for both valve types 16% and 96% for NVE 11% and 92% and PVE 19% and 98% [51]. In 2019, Chaosuwannakit et al. reported in 24 operated patients with 14 NVE and 10 PVE, the sensitivity and specificity of TTE were for both valve types 94.5% and 50%, for NVE 92.3% and 50% and PVE 87.5% and 33.3%. The sensitivity and specificity of CCT were for both valve types 94.1% and 66.67%, for NVE 92.3% and 50%, and PVE 85.7% and 66.67% [52]. In the same year, Hryniewiecki, et al. reported in 53 patients, with 58 NVE and 13 PVE, higher sensitivity and specificity of TTE (82% and 97%), lower in TEE (57% and 42%), while CCT was higher (89% and 71%). In the mutual application of all three diagnostic tests, the sensitivity was higher at 100% [53]. In 2020, Sifaoui et al. published the results in 68 operated patients with left-sided IE, with 15 PVE and 53 NVE. In the detection of 56 surgically confirmed vegetations, the sensitivity of TEE was 89.3% for both valve types, 91.1% for NVE, and 81.8% for PVE. The sensitivity of CCT was 80.4% in both valve types, 80% for NVE and 81.8% for PVE [54]. In 2020, Ye et al. published the results of 178 operated patients with the aortic valve IE with paravalvular spread. The sensitivity and specificity of CCT were 96% and 28%, while for TEE they were 100% [55]. In 2023, the latest study conducted of 78 patients, with 88 vegetation at 85 valves, reported slightly higher sensitivity for CCT (94%) vs. TTE (84.3%) and TEE (78.7%), but in mutual usage of all three imaging tests sensitivity was raised to 100% [31].

The first meta-analysis of the comparative diagnostic performance of TEE and CCT, published in 2020 by Oliveira et al., analyzed the results of eight available studies. In the detection of vegetation, the sensitivity and specificity of TEE were 94% and 82%, and for CCT were lower at 86% and 81% [56]. Another meta-analysis was published in 2021 by Jain et al., which analyzed 10 studies in 872 patients with IE. The sensitivity and specificity of TEE were higher at 96.2% and 83.1%, and for CCT were lower at 85.1% and 83.8% [57]. The aforementioned results are summarized in Table 3.

In NVE, CCT can better visualize vegetations in heavily calcified valves, especially in the form of a plate, in initially considered degenerative changes, or in severe destructed valves where vegetations are firmly attached, partially calcified, without oscillating movements, and considered part of the cusps. Also, CCT has better depiction of vegetation hidden in the commissural part, along the wall of the left ventricle outlet tract (LVOT), which is sometimes missed by echocardiography. CCT can make clear differentiation of vegetation from ruptured aneurysm. In PVE, due to metal reduction software, CCT can better depict vegetation, specifically of anterior localization. On the contrary, in right-sided endocarditis, CCT has limited visibility around pacemaker leads, when they present as coated thickening [31]. With some authors sensitivity correlates to the vegetation’s size. Ouchi et al., in 19 patients, detected a length of 6 mm as the cut-off value for vegetation detection [49]. In 68 patients, Sifaoui et al. found that a vegetation size of 7 mm was associated with the best pairing of sensitivity (66.1%) and specificity (75.0%) in CCT [54].

Differential diagnosis of vegetations includes thrombus, hypoattenuated leaflet thickening (HALT), fibroelastoma, Lambl’s excrescencies, and non-bacterial endocarditis (NBTE) [58,59,60,61,62]. Thrombus is irregular soft tissue lesion attached to the leaflets, sewing ring, or both, predominantly to the side of higher pressure [58]. In transcatheter aortic valve implantation (TAVI), HALT affects whole leaflets from the ring towards the free margin uniformly [59]. Echocardiography can depict thrombus as a mobile mass with signs of obstruction but cannot detect small thrombosis or thin HALT, where CCT is superior in localization and characterization [58,59]. Fibroelastoma are pedunculated lesions attached to the leaflet, rarely causing valve dysfunction or emboli due to thrombi adherence or disintegration [60]. Lambl’s excrescences are thinner filiform lesions that arise in commissural valve zones [61]. NBTE appears as small irregular lesions, commonly affects left-sided valves and is associated with malignancy, hypercoagulable states, and autoimmune disease (Libman–Sacks endocarditis). NBTE may be associated with embolism [62].

### 6.2. Aneurysms

Aneurysms appear as saccular outpouching of leaflets with loss of homogenous curvature (Figure 1 and Figure 2) [32,63]. They could be the only present IE-related lesion and are considered as a late complication. All aneurysms are localized in the direction of blood flow, and most often on the mitral valve, while the aortic and tricuspid valves are less common. Histologically, aneurysms consist of connective tissue containing remnants of the valve, lined with organized or fresh thrombotic masses. Often the tip of the aneurysm can perforate leading to worsening regurgitation [34,35,40].

In 2018, Kim et al. reported 100% agreement between TEE and CCT in aneurysm detection in five patients [64]. The latest study reported that in detection of 42 aneurysms confirmed at surgery, sensitivity for TEE was 31.6% whereas for CCT it was 100%, with average aneurysm depth 7.16 ± 2.65 mm and width 8.54 ± 4.25 mm [31].

### 6.3. Perforations

A perforation is loss of leaflet continuity, due to tissue necrosis caused by bacterial enzymes, and may be associated with severe valve regurgitation. Echocardiography with the additional use of color Doppler effect, detects two jet phenomena as a sign of perforation. CCT detects perforation as a defect that needs to be confirmed in longitudinal and transversal valve-planar reformation (Figure 2) [31,32].

Previous studies reported superiority of TEE over CCT in perforation detection, with sensitivity of TEE in the range 68.4–81.3% whereas for CCT at 41–68.4% [50,54,56]. In detection of 26 perforations, Kim at all reported agreement between TTE and CCT at 94.7% [64]. A recent study in the detection of 50 perforations confirmed at surgery, reported higher sensitivity of CCT 83.7% in comparison to TEE 63.9%. Better spatial resolution allows CCT to have better depiction of valve perforation in a hostile environment compared to echocardiography, such as heavily calcified valves in attachment zones. If there is more than one perforation, CCT is more sensitive in their overall detection [31]. CCT was superior if the perforations were localized in the adjacent cusps of the aortic valve or in the mitral valve, on the same leaflet, in nearby segments, or near the annulus. The technological progress of CCT has led to improved spatial resolution accompanied by software innovations. The use of double-oblique reformations enabled parallel observation of the valve in two planes, longitudinal and transverse, which increased the rate of visibility and certainty in the detection of perforations.

## 7. Cardiac CT Imaging of Paravalvular Lesions

### 7.1. Abscess

**Figure 4:**
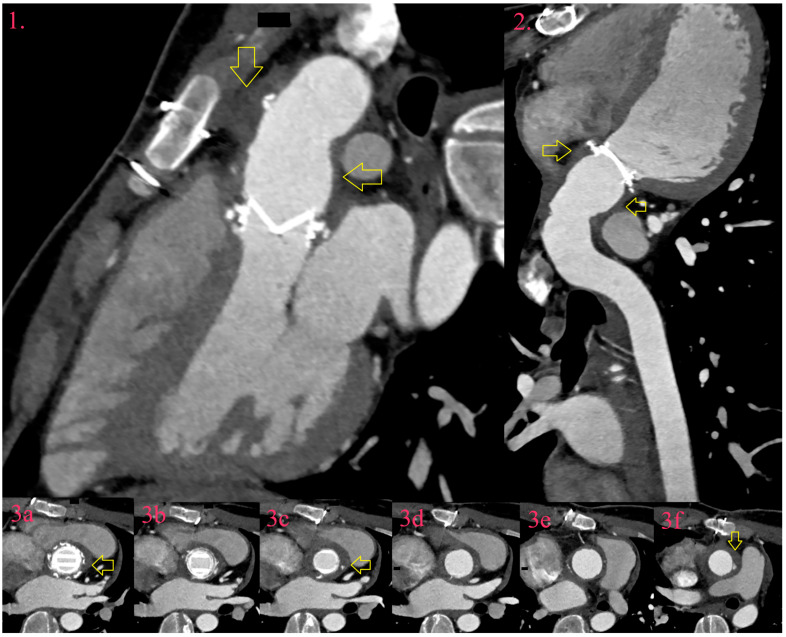
IE of artificial aortic valve and graft after Bentall procedure. There is asymmetric hypodense thickening around valve and graft in three-chamber view (Image 1, yellow arrow), curved planar reformation (Image 2, yellow arrow), and trans-axial images (Image 3a–f, yellow arrow).

An abscess is found in the perivalvular cavity with necrosis and purulent material and requires urgent surgical treatment. Echocardiography depicts an abscess as a non-homogenous hyperechogenic paravalvular thickening. CCT detects an abscess as thick hypodense paravalvular lesion clearly demarcated from surrounded fat, which converts the density of the perivalvular lipid layer from negative into positive values, approximately 20–50 HU (Figure 4) [63,65]. Hyperintense rim, as a sign of abscess capsule, is rarely seen in NVE, but in PVE, especially after surgery, is highly suspected to be active in inflammation rather than postoperative seroma [65].

An abscess develops from cellulitis and is defined by the localization and degree of expansion outside the annulus, determining its thickness, width, and percentage of involvement of the annular circumference. In NVE, the infection most often spreads through the subcommissural zone, while in PVE, the infection spreads along the suture ring, often encompassing the entire circumference. In the paravalvular tissue, bacteria perform enzymatic degradation of connective and fatty tissue, sparing muscle tissue. The spread of the abscess around the aortic native valve depends on the site of infection entry. If the process begins to spread between the right and left coronary cusps, it will extend towards the right ventricle outflow (RVOT). If it starts between the right and non-coronary cusps, it will spread towards the membranous part of the interventricular septum and also towards the right atrium and Koch’s triangle, destroying the atrioventricular node and the upper end of the bundle of His. If it starts in the subcommissural zone of the non-coronary and left coronary cusps, it will spread towards the aortomitral fibrosis and the LVOT. In the case of abscess of the native mitral valve, if the process spreads from the anterior leaflet abscesses will develop into aortomitral fibrosis. If the abscess starts from the posterior leaflet, it will spread along the atrioventricular groove and may lead to separation of the left atrium from the left ventricle. In PVE, the abscess most often covers the entire circumference of the ring. However, in the case of an artificial aortic valve, larger abscesses are often located posteriorly and to the left, below the trunk of the pulmonary artery. Pathways of abscess spread are useful knowledge in reading CCT examination, as the density between the abscess and the surrounded epicardial fat is slightly brighter, which may lead to false negative findings [35].

CCT is superior to TTE and TEE in abscess detection. Feuchtner et al. reported sensitivity and specificity for TEE of 89% and 100%, while CCT reached a sensitivity of 100% [45]. Gahide et al. reported the sensitivity and specificity of CCT at 100% and 87.5% [46]. Koo and et al. detected a higher sensitivity of CCT than TEE, 60% vs. 40% [48]. Also, Sims et al. detected higher sensitivity of CCT at 90.5% in comparison to TEE at 78.4% [50]. In PVE, Habets et al. reported the sensitivity and specificity of TEE at 68% and 91%, but when CCT was added to the diagnostic protocol, the sensitivity and specificity increased to 100% and 91%, [47] Sifaoui et al. detected rather similar sensitivity and specificity of CCT vs. TEE to be 77.3/72.7% vs. 72.7/89.1% [54]. But, Ye et al. reported, in 78 patients with aortic paravalvular IE, sensitivity and specificity of CCT 99/95% vs. TEE 86/91% [20]. In meta-analysis, Oliveira et al. detected sensitivity and specificity of CCT in abscess detection at 87% and 93% vs. TEE at 69% and 96% [56]. The latest research, in NVE and PVE, reported sensitivity of CCT in abscess detection to be 100% vs. 44.4% for TEE [31].

CCT is superior in abscess detection regardless of valve type and location, in comparison to echocardiography. In particular, CCT is better in detecting abscesses around surgical materials, around patches and grafts. In reading potential abscess around the grafts, the reader should keep in mind that hypodense thin rim <3 mm is considered normal finding due to graft material, but only if it is not in contact with nearby soft tissue collection towards anterior mediastinum [31].

### 7.2. Pseudoaneurysms

**Figure 5:**
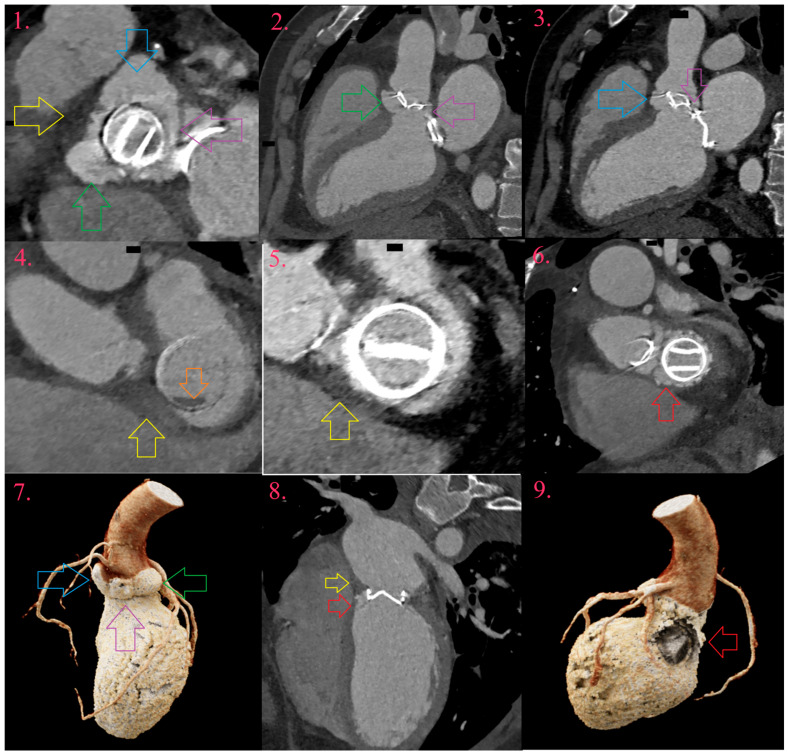
Multivalvular left-sided PVE. Around artificial aortic valve there is pseudoaneurysm abutting its entire circumference (Image 1, Image 2, and Image 7, green arrow), with development of two fistula between aorta and left ventricle, through left coronary sinus (Image 1, Image 3 and Image 7, blue arrow) and aortomitral fibrosis (Image 1, Image 2, Image 3 and Image 7, purple arrow). There is abscess towards right ventricle outflow tract (Image 1, yellow arrow) and right fibrous trigonum (Image 4, Image 5, and Image 8, yellow arrow), with development of pseudoaneurysm around artificial mitral valve in left ventricle outflow tract (Image 6, Image 8, and Image 9, red arrow). At the tip of the artificial mitral valve, there is vegetation toward left atrium (Image 4, orange arrow).

A pseudoaneurysm is a drained abscess which communicates with the cardiac chamber or aorta [32]. As abscess, it is defined by localization and degree of expansion outside the annulus, determining the depth and size of the cavity, and the percentage of the involvement of the annular circumference. It occurs almost exclusively in left-sided IE, probably due to high blood pressure and oxygenated blood. In NVE, it usually begins in the subcommisural zone, where it expands from a small point of entry into the paravalvular space. In PVE it usually begins from the sewing ring and spreads along the valve circumference (Figure 5) [40]. Echocardiography depicts pseudoaneurysm as pulsatile cavity with color Doppler flow. CCT depicts pseudoaneurysm as contrast filled paravalvular cavity, pulsatile on cine images [32,63,65]. TTE may visualize pseudoaneurysm in the anterior position and to the right side, whereas TEE is superior in the detection of pseudoaneurysm in the posterior position, to the left side and in intervalvular fibrosa. But with lower spatial resolution, artifacts, due to heavy calcification of the valve annulus or vessel wall or metal artifacts from prosthetic material, diminish detection of small pseudoaneurysm as well as the depth and circumferential extension of larger ones [37]. All the abovementioned obstacles are surpassed by adding CCT to the IE imaging protocol due to better spatial resolution. Localization of pseudoaneurysms and pathways of their spread are at the place of the previously described abscess collections.

Gahide et al. reported sensitivity and specificity of CCT in detection of pseudoaneurysms as 100% and 87.5%, in aortic IE in 19 patients [46]. Sifaoui et al., in 68 patients, reported sensitivity and specificity of CCT in pseudoaneurysm detection as 100% and 96.8% in comparison to TEE 72.7% and 89.1% [54]. The majority of studies report mutual sensitivity and specificity of CCT in detection of abscesses and pseudoaneurysms in a range 60–100% and 78.4–100% vs. TEE 40–92.3% and 89–100% [45,48,50,51]. CCT depicts pseudoaneurysms independent of their localization, accurately assesses depth and size of its cavities and the percentage of the circumference of the annulus involved, information which is relevant to the surgeon [31].

### 7.3. Fistula

A fistula is communication between two neighboring cavities through an abnormal penetrating tract, usually as a consequence of an abscess or pseudoaneurysm, and associated with poor clinical outcome [63]. It is a channel lined with thrombotic masses, with the possible presence of microorganisms. Abscesses around the aortic valve can form fistulas between the ascending aorta and any heart chamber, but most often left-sided. Abscesses around the mitral valve can create a fistulous channel between the left atrium and ventricle, independent of the orifice [33,35,40].

Echocardiography depicts fistula as paravalvular color-Doppler flow between two cavities. CCT depicts fistula as paravalvular contrast-filled tunnel between two neighboring cavities (Figure 4) [63].

Sims et al. reported lower sensitivity and specificity of CCT in fistula detection as 50% and 96.9% vs. TEE 78.6% and 98.7%, in 34 operated patients with preoperative CCT [50]. Ye et al., in 178 patients with aortic paravalvular IE, reported similar specificity and sensitivity for CCT as 100%/98% and for TEE 100%/96% [55]. The latest meta-analysis of Jain et al. reported higher sensitivity and specificity of CCT at 98%/90% in comparison to TEE at 85.7%/98.6% [57].

CCT provides additional information to TEE in the precise depiction of fistula morphology and dimensions due to better spatial resolution, especially in PVE [31].

### 7.4. Leak

Destruction of the prosthetic valve ring leads to valve dehiscence and paravalvular leak. Echocardiography depicts leak as paravalvular color Doppler flow, with or without rocking motion of the prosthesis [32,63]. CCT depicts leak as thin contrast-filled cavity near the prosthetic valve clearly differentiated from the sewing ring. TEE is more sensitive in leak detection in comparison to CCT. Koo et al. reported lower sensitivity of CCT in leak detection at 50% in comparison to TEE at 100% [48]. Also, two available meta-analyses reported lower sensitivity and specificity of CCT in comparison to TEE; Oliveira et al. reported for CCT 72%/69% vs. TEE 100%/99%, whereas Jain et al. reported for CCT 85.1%/100% vs. TEE 100%/95.6% [56,57]. With development of the software for reduction of metal artefacts, there is improvement in leak detection by CCT, but the reader should keep in mind the difference between sewing ring and leak, in order to prevent false positive findings [31].

## 8. Cardiac Computed Tomography Angiography

Initially, CCT was introduced into the diagnostic algorithm of infective endocarditis, in order to evaluate anatomy and coronary artery disease, in the case of aortic valve involvement, in order to avoid a high embolic risk, when performing invasive coronary angiography. Coronary artery analysis is recommended if the patient is a candidate for cardiac surgery. The European Association of Cardiology, in the latest published guidelines for the treatment of infective endocarditis in 2023, recommended preoperative coronary angiography for men older than 40 years, postmenopausal women, and in the population with one or more risk factors or a history of coronary artery disease. Alternatively, coronary computed tomography angiography is used to rule out significant coronary obstruction [19]. Clinical application is supported by the high sensitivity and negative predictive value (>95%) of this imaging method to rule out coronary artery disease, especially in low- to intermediate-risk patients. One of the disadvantages of this method is the overestimation of the severity of stenosis in highly calcified plaque, especially in medium stenosis, which can be an advantage in making the decision for cardiac surgery [66,67] Recent studies questioned the necessity of coronary artery bypass grafting of non-critical lesions during surgery of IE as it could have a negative impact on peri-operative outcome [19].

## 9. Left-Sided Infective Endocarditis

### 9.1. Mitral Valve Infective Endocarditis

The mitral valve is affected in 40% of all cases of infective endocarditis. Patients with valve prolapse and degenerative annular calcification are at the highest risk. Along with echocardiography, CCT can be useful in the precise localization of IE lesion, which plays an important role in the decision-making process of the appropriate surgical treatment—valve repair versus valve replacement. As the feasibility of valve repair depends on the extent of tissue destruction, patients with limited valvular infection are the best candidates. On the other hand, extensive damage of the anterior leaflet, large lesions involving the posterior leaflet or commissures, and annular abscesses are considered the main obstacles to mitral repair. Specifically, when the vegetation is localized on the posterior leaflet at the P1 segment or P3 segment and on the posteromedial commissure, reconstruction after excision of the infected segment can be performed, with a limited resection of this scallop. CCT is a useful imaging method in detection of valvular IE lesions, especially around heavy calcification. CCT is a superior imaging method in detection of annular abscesses, and their involvement of fibrous trigonums and aortomitral fibrosis, which is usually missed with echocardiography [68].

### 9.2. Aortic Valve Infective Endocarditis

Initially, CCT was introduced in the field of imaging of infective endocarditis, for the assessment of anatomy and coronary artery disease in patients with aortic valve infective endocarditis. Until now, its indication area has been significantly expanded. In aortic NVE, patients with bicuspid aortic valve (BAV) and degenerative valve disease are at the highest risk. CCT is a useful imaging method in confirming the type of BAV, especially in cases of partial fusion of cusps. In detection of valvular lesions, CCT is useful in depicting small aneurysms and uniform thickening of valve cusps, whereas detection of small vegetations depends on its thickness and image quality. In detection of paravalvular lesions, both NVE and PVE, CCT is a superior imaging method. In the last 17 years, transcatheter aortic valve implantation (TAVI) has revolutionized the treatment of aortic stenosis, leading to an expanded population with prosthetic valves. Cahill et al. reported that the risk factors for TAVI IE in the study cohort included younger age, male sex, atrial fibrillation, and dialysis. CCT is useful in differential diagnosis of IE from hypo-attenuated leaflet thickening in patients with TAVI and embolic events. CCT may differentiate vegetation from uniform leaflet thickening, from attachment to the free edge, such as seen in HALT [36,69,70].

## 10. Right-Sided Infective Endocarditis

Incidence of right-sided infective endocarditis accounts for 5–10% of all cases. The lower incidence can be explained by the relatively low prevalence of pathological conditions affecting right-sided valves, differences in the properties, and vascularity of the right-sided endothelium, as well as lower pressure gradients and jet velocities across the right-sided valves, lower right-sided wall stress, and reduced oxygen content in venous blood. TTE provides sufficient information due to the proximity of the right heart chambers to the chest wall, but its deformities and chronic obstructive pulmonary disease may limit examination. TEE is in the proximity of the left cardiac chambers, and sometimes cannot provide sufficient information. Cardiac CT may surpass these obstacles, but with a correct tailored protocol. Turbulent flow of a mixture of saline and contrast agents forms superior vena cava to the right heart chambers, degrades homogenous contrast distribution and valve visualization, which could be overcome by slower flow rates. In treatment of right-sided endocarditis, antibiotic therapy is the cornerstone. Surgery is considered after failure of medical therapy, for large vegetations, recurrent septic pulmonary emboli, and infected prosthetic valves. Surgical techniques consist of vegetation removal and valve repair, rather than valve replacement or ring annuloplasty, as the use of prosthetic material should be avoided when possible [71].

### 10.1. Tricuspid Valve Infective Endocarditis

In right-sided infective endocarditis, 90% of cases involve the tricuspid valve. It is usually associated with intravenous drug use (IVDU), intracardiac devices, indwelling venous catheters (hemodialysis, parenteral nutrition, and chemotherapy), and patients with congenital heart disease. The predominant causative microorganism is Staphylococcus aureus. CCT usually depicts vegetations attached to the leaflets, which can be large, whereas perforation and aneurysm are rare. Paravalvular extension beyond the annulus, such as the formation of abscesses, pseudoaneurysms, or fistula, is rare and accounts for 0.7% of cases. The process could spread from left-sided infective endocarditis, with extension of aortic root abscesses or with ventricular septal defects as IE favors the right heart (lower pressure) side of the defect. It is depicted by thickening of the atrial and membranous part of the interventricular septum, with formation of vegetation, and is associated with worse prognosis. Also, infection could spread from pacemaker wires, where CCT has difficulties in depicting vegetation presented by coated thickening around wires due to metal artefacts, but it could easily depict in the form of verruca. Nonfunctional embryonic remnants that are present in the right atrium, such as crista terminalis, eustachian valve, Chiari network, and Thebesian valve, can mimic vegetation, but they can also be a nidus for IE. The prevalence of IE affecting these structures is thought to be underreported and underestimated because of the difficulty with detection by TTE as well as the paucity of reports describing them, but in suspected cases CCT could be a problem-solving tool. After central venous catheter removal, a fibrin sheath, a circumferential sleeve of endothelium formed around its external surface, remains in SVC after its removal and could be infected, with propagation to the right atrium and tricuspid valve [71,72].

### 10.2. Pulmonary Valve Infective Endocarditis

Pulmonic valve IE may occur concomitantly with tricuspid valve IE. Isolated pulmonic valve IE is rare and accounts for <2% of patients with IE. It is usually associated with congenital heart disease; isolated, such as bicuspid, unicuspidal or dysplastic valve, or, complex, such as univentricular heart, tetralogy of Fallot (TOF), and double outlet right ventricle (DORV). The consequence is the development of pulmonary stenosis as the culprit lesion. Patients with valve conduits used for the restoration of right ventricle-to-pulmonary artery (RV-PA) continuity are at higher risk of late IE. Pulmonary homograft, porcine heterograft (Hancock bioprosthetic valve conduit; Medtronic), and bovine jugular graft (Contegra pulmonary valve conduit; Medtronic, Minneapolis, MN, USA) are used, the latter of which is at the highest risk. Also, percutaneous pulmonary valve replacement is becoming more common. McElhinney et al. reported on the prevalence of IE in 309 patients, who underwent transcatheter pulmonary valve replacement, with a Melody valve. Usually, TTE could not provide sufficient information, in which cases CCT is a useful method to confirm and/or to detect new IE lesions missed by echocardiography. In pulmonary valve NVE, CCT is a useful tool in depicting valve type; valvular lesions, of which vegetations is the most common, at the ventricular side of the cusps; and paravalvular lesions, usually in form of abscess near commissural parts, but rarely seen. In cases of IE of pulmonary conduits, CCT is a superior method in detection of graft infection. But, in detection of valvular lesion in PVE, CCT is similar to TTE due to metal artefacts, and superior in detection of paravalvular lesions [71,73,74,75].

## 11. Systemic Embolism in Infective Endocarditis

Embolic events are frequent and potentially life-threatening complications of IE due to migration of cardiac vegetations [19]. Whole-body CT is a usuful imaging method in detection of systemic embolism, but there are no clear recommendations defining the setting in which it should be used [32]. 2023 ESC guidelines for the management of endocarditis are engaged in predicting the risk of embolism and the use of the appropriate imaging method once the complications have clinically occurred [19].

### 11.1. Neurologic Complications

Symptomatic neurologic complications occur in 35% patients, whereas in 80% of patients they remain clinically silent. They include ischemic stroke, transient ischemic attack, hemorrhage (intracerebral, subarachnoid), meningitis, brain abscess, encephalopathy, and infectious aneurysm. Evaluation should include MRI with or without gadolinium, or CT with or without contrast if MRI is not possible. Catheter angiography should be performed in patients with infective aneurysm, in acute brain hemorrhage, with suspicion of aneurysm despite negative non-invasive imaging or if mechanical thrombectomy is considered [19,32].

### 11.2. Thoracic Complications

**Figure 6:**
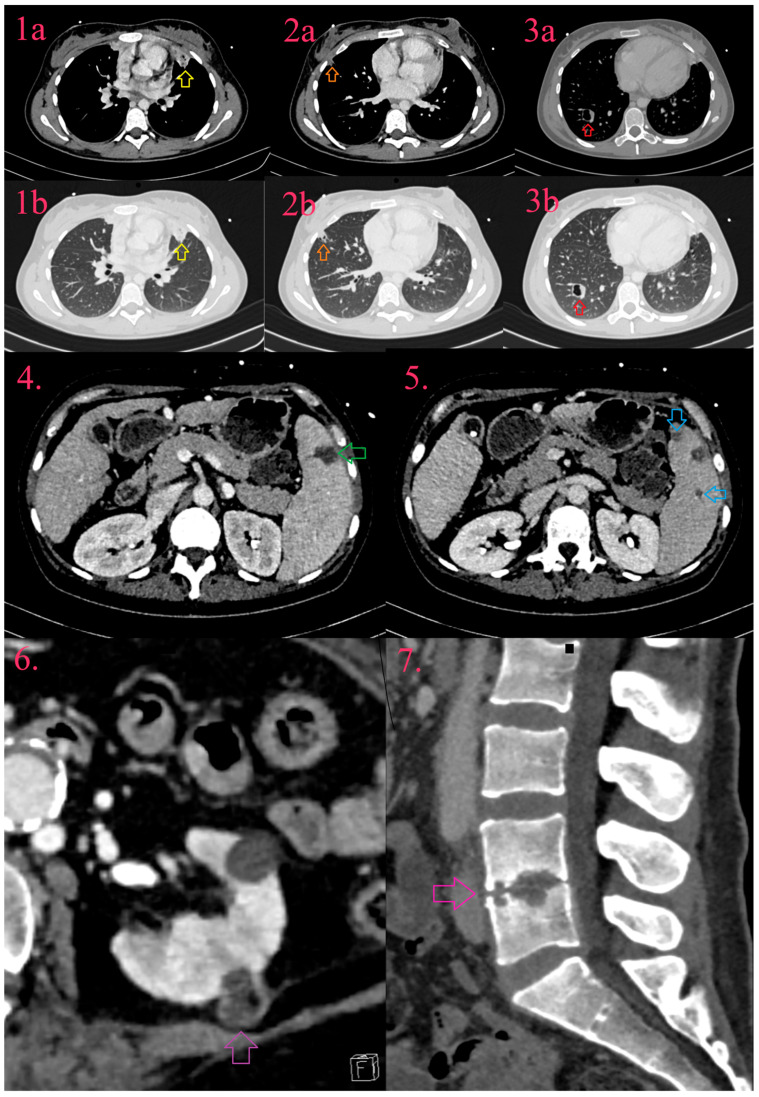
Systemic dissemination of infective endocarditis. Multiple lung abscesses in form of consolidation (Image 1a and 1b, yellow arrow), partial cavitation (Image 2a and Image 2b, orange arrow), and cavitation with fluid level (Image 3a and Image 3b, red arrow). Spleen abscess (Image 4, green arrow) and two infarcts (Image 5, blue arrow). Abscess of left kidney (Image 6, purple arrow). Spondylodiscitis with loss of intervertebral space and erosion of end-plates of vertebral bodies L4 and L5 (Image 7, pink arrow).

There are almost exclusively consequences of right-sided IE and they occur in 14% of patients with IE with systemic embolism. They include pulmonary infarction, pulmonary abscess, pleural effusion, and empyema (Figure 6, Images 1a,b, Images 2a,b and Images 3a,b) [19,32].

### 11.3. Vascular Complications

Mycotic aneurysms have rare complications defined as infections of the vascular wall. They are more frequent in the central nervous system, abdominal aorta, and superior mesenteric artery, but in 6% of cases may affect coronary arteries. CT depicts them as segmental saccular pseudoaneurysms [32,76].

### 11.4. Abdominal Complications

Abdominal complications are associated with left-sided infective endocarditis, with the spleen as the most often affected organ (19–32%), following the kidneys (6–14%), and rarely the liver (3–11%) [76]. Splenic involvement includes asymptomatic infarction, with progression to abscess and potential rupture (Figure 6, Image 4 and Image 5) [19]. The kidney follows the same development pattern as in the spleen; infarcts progress to abscesses and are often multiple, sometimes bilateral (Figure 6, Image 6). Liver involvement is manifested by abscess. CT depicts infarct, independent of the affected organ, as triangular hypodense lesion, which with the development of contrast-enhanced peripheral rim, develops into abscess, that may strain the organ capsule [32].

### 11.5. Musculoskeletal Complications

Metastatic bone or joint-IE related lesions are relatively frequent, and include spondylodiscitis (2–10%), osteomyelitis, and septic arthritis. MRI is a preferable imaging modality in comparison to CT. In spondylodiscitis, CT detects indirect signs of disease such as loss of disc height, erosion or destruction of end-plates and vertebral bodies, and paravertebral soft tissue collections with abscess development (Figure 6, Image 7) [19,32].

## 12. Limits of Cardiac Computed Tomography and Gaps in Evidence

Patients suffering from infective endocarditis are often characterized by an irregular heart rhythm and high heart frequency (above 90 s per minute), which, despite the technical improvement of CT machines, greatly degrades the spatial resolution of the image and the accuracy of its reading. CT machines provide lower temporal resolution in comparison to echocardiography, which is a useful feature in the assessment of oscillatory movements of IE lesions. The patients are exposed to ionizing radiation.

The knowledge of the diagnostic performance of CCT, available so far, is based on the results of less than fifteen comparative studies, between CCT and echocardiography, with a variable number of participants (only three studies had more than 100 participants) and methodology. Among which there are large discrepancies in the diagnostic performance of imaging techniques in the detection of valvular lesions, whereas there is solid agreement in the detection of paravalvular lesions. There is a need for a greater number of comparative studies in the detection of precisely defined valvular IE lesions, and potential answers to the following questions:

- What is the threshold thickness and length of vegetation for visibility on CCT?
- What is the real diagnostic performance of CCT in the detection of aneurysms, as a late complication in relation to echocardiography and operative findings?
- What is the real diagnostic performance of CCT in the detection of perforation in comparison with echocardiography?

## 13. Future Directions of CCT in Infective Endocarditis

Further technological improvement will have a significant impact on improved spatial resolutions and acquisition of the heart in almost one cardiac revolution, which will make it less susceptible to artifacts of irregular heart rhythm and high heart frequency. This will influence the situation in that more future diagnostic comparative studies will confirm the diagnostic accuracy of CCT in the detection of valvular lesions of IE, which will increase the use of this imaging method and make it inevitable in daily practice. 

## 14. Conclusions

Cardiac CT has emerged as a powerful diagnostic imaging method in the evaluation of morphology of IE lesions. As an additive diagnostic imaging method, it confirms the existence of suspicious IE lesion during echocardiography. As a complementary diagnostic imaging method, it detects the IE lesions that were missed during the echocardiographic examination. Therefore, cardiac CT can be assumed to be the main diagnostic imaging modality due to its superior spatial resolution. However, the lack of hemodynamic assessment and low temporal resolution classify it as an additive and complementary diagnostic imaging method in the regular clinical routine for patients suffering from infective endocarditis. Cardiac CT is recommended in patients with possible NVE to detect valvular lesion and in suspected NVE and PVE to diagnose paravalvular lesion. Cardiac CT in conjunction with echocardiography, transthoracic and transesophageal, improves the accuracy and timeliness of diagnosis. Consecutive CT of thorax, abdomen, and pelvis allows prompt detection of systemic manifestation of IE and appliance of appropriate therapy.

## Figures and Tables

**Table 1:** Comparison of the single cardiovascular imaging techniques in the diagnosis of IE.

Imaging Modalities	Advantages	Limitations
Echocardiography	Good diagnostic ability in detection of valvular lesions High temporal resolution and detection of oscillatory movement of vegetation color Doppler options and detection of small perforation in NVE and dehiscence around PVE Valvular function assessment Useful in monitoring therapy response Useful for follow-up	Lower spatial resolution Limited diagnostic ability in detection of paravalvular lesion Limited in detection of anterior cardiac structures and RVOT in right-sided IE Unable to detect systemic embolism Periprocedural complications in transesophageal echocardiography
Cardiac CT	Good diagnostic ability in detection of valvular lesions Superior diagnostic ability in detection of paravalvular lesions Higher spatial resolution Coronary artery assessment Assessment of systemic embolism	Lower temporal resolution Limited detection of thin vegetations Radiation exposure Risk of nephrotoxicity
PET/CT	Superior diagnostic ability in PVE Provides combination of metabolic activity and morphology of IE lesion	Low diagnostic ability in NVE, especially in vegetation detection Low temporal and spatial resolution False positive results due to inflammation up to one year after surgery Patient preparation Radiation exposure Risk of nephrotoxicity
WBC SPECT/CT	High specificity for infection	Low diagnostic ability Low spatial resolution Lower acquisition time Radiation exposure

CCT—cardiac computed tomography; PET/CT—positron emission tomography/computed tomography; WBC SPECT/CT—white blood cell single photon emission tomography/computed tomography.

**Table 2:** Comparative description of IE-related lesion at surgery, echocardiography, and cardiac CT.

IE Lesion	Surgery/Autopsy	Echocardiography	Cardiac CT
Vegetation	Infected mass attached to an endocardial structure or on implanted intracardiac material	Oscillating or non-oscillating intracardiac mass on valve or other endocardial structure, or on implanted intracardiac material	Hypodense mass attached to the valve, endocardium or prosthesis
Aneurysm	Saccular outpouching of valvular tissue	Saccular outpouching of valvular tissue	Saccular outpouching of valvular tissue
Perforation	Interruption of endocardial tissue continuity	Interruption of endocardial tissue continuity traversed by color Doppler flow	Interruption of endocardial tissue continuity confirmed in two planes
Abscess	Perivalvular cavity with necrosis and purulent material not communicating with cardiovascular lumen	Thickened, non-homogenous perivalvular area with echodense or echoluscent appearance	Hypodense zone with a vascular rim. Soft tissue thickening around the valve and great blood vessels as a sign of early abscess
Pseudoaneurysm	Perivalvular cavity communicating with the cardiovascular lumen	Pulsatile perivalvular echo-free space with color Doppler flow detected	Perivalvular contrast-filled cavity
Fistula	Communication between two neighboring cavities trough a perforation	Color Doppler communication between two neighboring cavities trough a perforation	Contrast-filled communication between two neighboring cavities trough a perforation
Leak	Dehiscence of prosthesis	Paravalvular regurgitation identified with color Doppler with or without rocking motion of the prosthesis	Malalignment of the prosthesis and annulus with tissue defect. Rocking motion of more than 15° on cine CT images

**Table 3:** Study correlation of diagnostic performance of echocardiography and CCT in detection of vegetations.

Authors	Year	Study Type	No of Participants	No of Valves	Valves Types	Diagnostic Performance of TEE	Diagnostic Performance of CCT
Feuchtner et al. [45]	2009	Retrospective	29	57	NVE + PVE	Sn 97% Sp 95%	Sn 96% Sp 97%
Gahide et al. [46]	2010	Prospective	19	19	NVE + PVE	NR NR	Sn 71.4% Sp 100%
Habets et al. [47]	2013	Prospective	28	28	PVE	Sn 63% Sp NR *TTE + TEE	Sn 100% Sp NR *+CCT
Koo et al. [48]	2017	Retrospective	49	47	NVE + PVE	Sn 100% Sp NR	Sn 90.9% Sp NR
Ouichi et al. [49]	2018	Retrospective	14	14	NVE + PVE	Sn 100% Sp NR	Sn 92.3% Sp NR
Sims et al. [50]	2018	Retrospective	255	NR	NVE + PVE	Sn 95.6% Sp 93%	Sn 70% Sp 92.9%
Koneru et al. [51]	2018	Retrospective	122	141	NVE + PVE	Sn 85% Sp 69%	Sn 16% Sp 96%
Chaosuwannakit et al. [52]	2019	Retrospective	24	24	NVE + PVE	Sn 94.5% Sp 50% *TTE	Sn 94.1% Sp 66.67%
Hryniewiecki et al. [53]	2019	Prospective	53	71	NVE + PVE	Sn 57% Sp 42%	Sn 89% Sp 71%
Sifaoui et al. [54]	2020	Prospective	68	68	NVE + PVE	Sn 89.3%	Sn 80,4%
Ye et al. [55]	2020	Retrospective	178	NR	NVE + PVE	Sn 100% Sp 100%	Sn 96% Sp 28%
Oliveira et al. [56]	2020	Meta-analysis (8 studies)	336	NR	NVE + PVE	Sn 94% Sp 82%	Sn 86% Sp 81%
Jain et al. [57]	2021	Meta-analysis (10 studies)	872	NR	NVE + PVE	Sn 96.2% Sp 83.1%	Sn 85.1% Sp 83.8%
Petkovic et al. [31]	2023	Retrospective-prospective	78	85	NVE + PVE	Sn 78.7% Sp 25%	Sn 94% Sp 60%

Sn—Sensitivity; Sp—Specificity; *TTE + TEE—Diagnostic performance corresponds to the mutual usage of both test; *+CCT—Diagnostic performance corresponds to the mutual usage of TEE and CCT; *TTE—Diagnostic performance refers only to the TTE, as TEE wasn’t analyzed.

## Data Availability

The data presented in this study are available upon reasonable request from the corresponding authors.
